# Traumatic Venous Sinus Thrombosis: Patient and Practice Patterns at a Major Trauma Center

**DOI:** 10.1007/s12028-025-02278-1

**Published:** 2025-05-22

**Authors:** Deborah L. Huang, Ritwik Bhatia, Rubinee Simmasalam, Jason F. Talbott, Michael C. Huang, Vineeta Singh

**Affiliations:** 1 Department of Neurology, University of California San Francisco, San Francisco, CA USA; 2 Department of Radiology, Zuckerberg San Francisco General Hospital, San Francisco, CA USA; 3 Department of Neurosurgery, Zuckerberg San Francisco General Hospital, San Francisco, CA USA; 4 Department of Neurology, Zuckerberg San Francisco General Hospital, San Francisco, CA USA

**Keywords:** Traumatic brain injury, Intracranial sinus thrombosis, Cerebral veins, Traumatic cerebral hemorrhage, Treatment outcome

## Abstract

**Background:**

Traumatic brain injury can lead to venous sinus injury and thrombosis, which may be associated with elevated intracranial pressure and poor outcomes. We sought to examine the risk factors, management, and clinical outcomes of traumatic venous sinus thrombosis (tVST).

**Methods:**

We conducted a comprehensive search of our institutional radiology database for final radiology reports from 2013 to 2022 that contained the terms “venous sinus thrombosis,” “sinus thrombosis,” or “venous occlusion.” tVST was detected on computed tomography and confirmed by a board-certified neuroradiologist.

**Results:**

We identified 135 patients on initial screening and entered 112 into our final analysis. Patients were predominantly male (76.8%) and had a mean age of 44 years. Initial Glasgow Coma Scale scores of 13–15, 9–12, and 3–8 were found in 60.7%, 12.5%, and 26.8% of our cohort, respectively. Eighty-nine patients (79.5%) were alive at hospital discharge. Most patients sustained skull fractures (*n* = 109, 97.3%), including skull base fractures. Seventeen patients required interventions for refractory intracranial hypertension, of whom 16 (94.1%) had multiple tVST. We observed heterogeneity in tVST monitoring and treatment practices. Patients received anticoagulation (AC; 13.4%), antiplatelet (AP; 34.8%), or conservative (no AC or AP; 51.8%) treatment for tVST. Follow-up imaging was available for 52 patients, showing recanalization of venous sinuses in 26 patients (50%) by 6 months post injury. Recanalization rates were higher in the AP group than in the AC group. However, this was likely the result of selection bias, in which patients with mild to moderate injuries were more likely to be assigned to AP therapy. We noted more bleeding complications in AC- and AP-treated patients (20.0% and 12.8%) than in conservatively managed patients (3.4%), even after adjusting for lower survival in the conservative group.

**Conclusions:**

Differences between treatment groups should be cautiously interpreted due to selection bias and confounding by indication. More studies are needed to determine the optimal management of tVST.

## Introduction

Traumatic brain injury (TBI) results in more than 200,000 hospitalizations and 60,000 deaths in the United States every year [1]. The global incidence of TBI is an estimated 69 million people per year and spans patients of all ages [2]. In addition to pain and suffering for patients and their families, the economic impact of nonfatal TBI in the United States is estimated at US $40–76 billion per year [1–3]. The prognosis and recovery timeline for TBI varies widely, depending on factors such as TBI severity, lost functionality, and other polytrauma related injuries. Patients with TBI are at risk for secondary brain injury because of infections, seizures, fevers, volume depletion, and cerebrovascular injuries such as arterial dissection, vasospasm, and venous sinus thrombosis (VST).

Traumatic VST (tVST) is increasingly detected on neuroimaging, particularly after certain skull fracture patterns [4, 5]. Screening is crucial in patients with TBI, as VST symptoms of progressive headache, vomiting, seizures, impaired consciousness, and focal neurologic deficits may be attributed only to the primary brain injury [6]. VST can contribute to elevated intracranial pressure (ICP) and poor outcomes, highlighting the importance of timely detection and appropriate management [7, 8]. Current guidelines recommend treatment of spontaneous VST with therapeutic anticoagulation (AC), but the optimal treatment of tVST remains unclear [9]. As a result, diagnostic and treatment practices vary widely. Supportive management of tVST includes hydration, hyperosmolar therapies, and anticonvulsants [5]. Case reports and case series have reported mixed findings regarding the safety and necessity of anticoagulating patients with tVST, making antithrombotic treatment of tVST controversial [10–13]. Studies investigating the management and outcomes of tVST have been limited by small sample sizes and nonrandomized study designs. We examined the risk factors, management strategies, and clinical outcomes of tVST within our large urban trauma center in San Francisco, California.

## Methods

A retrospective review of all patients who arrived at our level one trauma center between December 2013 and December 2022 was conducted. Using mPower Clinical Analytics software from Nuance (Burlington, MA), we performed a comprehensive search of our entire institutional radiology database. Radiology reports were queried for the terms “venous sinus thrombosis,” “sinus thrombosis,” and “venous occlusion.” Venous sinus thrombosis detected on computed tomography (CT) was confirmed by a board-certified neuroradiologist. Patients with venous sinus narrowing without thrombosis and VST not related to trauma were excluded. This study was approved by the institutional review board and deemed exempt from obtaining patient consent.

Patients who arrived at our emergency department with TBI and loss of consciousness or posttraumatic amnesia underwent a noncontrast CT scan of the head if any of the following were present: headache, vomiting, age > 60 years, drug or alcohol intoxication, deficits in short-term memory, posttraumatic seizure, Glasgow Coma Scale (GCS) score < 15, focal neurologic deficit, coagulopathy (international normalized ratio > 1.4), active use of AC or dual antiplatelet therapy (DAPT), or physical evidence of trauma above the clavicle. A noncontrast CT scan of the head was considered for patients with TBI who did not have loss of consciousness or posttraumatic amnesia if the following were present: focal neurologic deficit, vomiting, severe headache, age > 65 years, physical signs of a basilar skull fracture, GCS score < 15, coagulopathy (international normalized ratio > 1.4), active use of AC or DAPT, or dangerous mechanism of injury (ejection from a motor vehicle, pedestrian vs. auto, or fall from height greater than three feet or five stairs).

Both blunt and penetrating head injury patterns were included in this study. If patients were found to have calvarial fractures that involved the skull base, a carotid canal, or major venous sinus, they underwent craniocervical vascular imaging with CT venography (CTV) or CT angiography (CTA) of the head with delayed contrast phase to evaluate for venous sinus pathological findings. A minimum Hounsfield Units value of 175 for the sigmoid sinus density was confirmed on each CTA with delayed contrast timing to ensure that the venous evaluation was adequate. For details regarding image acquisition protocols and modality selection (acquisition parameters and situations when CTA was used in lieu of CTV), please refer to the methods described by our group in a separate publication on the classification of traumatic venous sinus injuries [14]. Follow-up imaging of the venous sinuses was performed with either CT or magnetic resonance (MR) imaging with venous-phase timed contrast.

Following initial scanning and stabilization in the emergency department, patients with TBI were admitted either to our neurosurgical intensive care unit or neurosurgical ward. Patients received 7 days of prophylactic anticonvulsant therapy if imaging showed intracranial hemorrhage beyond the subarachnoid space. Patients underwent continuous electroencephalography monitoring if the GCS score was < 9 or at the discretion of the treating physician. Electrographic and clinical seizures were treated with escalation of anticonvulsant therapy at the discretion of the attending physician on service. The decision to place an extraventricular drain (EVD) and/or parenchymal ICP monitor was made by the neurosurgical attending physician. Patients with ICP > 20 mm Hg for at least 5 min received hyperosmolar therapy and standard ICP-lowering measures when indicated. Decompressive hemicraniectomy was performed as needed for hematoma evacuation and/or as a third-tier therapy for refractory ICP elevation, which was defined as ICP > 20 mm Hg for at least 15 min despite administration of first-tier and second-tier medical therapies outlined by the Brain Trauma Foundation [15].

The management of tVST for each patient was discussed in multidisciplinary rounds between both neurosurgery and neurocritical care teams. The choice of treatment strategy with full systemic AC, antiplatelet (AP) medication, or conservative management (no AC/AP) was left to the discretion of the attending physician on service. We did not have an institutional protocol to guide the timing of initiation, dosing, or duration of AC and AP therapy. Antiplatelet medication consisted of either 81 mg of aspirin, 325 mg of aspirin, or 75 mg of clopidogrel. Patients who were treated with AC were first started on an unfractionated heparin infusion and then switched to weight-based low-molecular-weight heparin or a direct oral anticoagulant. The duration of therapy was left to the prescribing physician’s discretion. In most cases, AC was continued for at least 3 months. Low-dose enoxaparin for deep vein thrombosis (DVT) prophylaxis was initiated 48 h after bleed stability or as soon as the neurosurgical team felt this was safe. The protocolization of DVT prophylaxis in trauma patients at our institution occurred midway through the data set timeline. We did not classify DVT prophylaxis as antithrombotic therapy in our analysis.

Through manual chart reviews, we collected demographic information, mechanism of injury, GCS score at the time of hospital admission, and past medical history, including prearrival antithrombotic use. We obtained radiologic data regarding skull fractures, VST, and intracranial hemorrhages. If repeat venous imaging was available, we also collected information on tVST improvement (as noted by the search terms “improved,” “improvement,” “resolution,” or “recanalization”) or lack thereof (as noted by the search terms “worsened,” “propagation,” “extension,” “no improvement,” “persistent,” or “no change”). The timing and modality of repeat venous imaging was not standardized. We noted the discharge neurologic examination, seizures, leukocytosis, and interventions that were used to treat elevated ICP (hyperosmolar therapy, surgical decompression, cerebrospinal fluid diversion). We recorded intravenous fluid administration, anticonvulsant use, antithrombotic regimen (AC or AP), hospital length of stay (LOS), discharge disposition, withdrawal of life-sustaining treatment (WOLST), and bleeding complications. Clinical outcomes were gathered from notes within the index admission, Epic CareEverywhere records, and subsequent hospital admissions.

Normally distributed continuous variables were summarized descriptively using means standard deviations, and nonnormally distributed variables were summarized descriptively using medians (interquartile ranges [IQRs]). Categorical variables were summarized with counts and percentages. We used Cox regression models to analyze associations between age, sex, GCS score, hospital LOS, tVST treatment strategy, time to bleeding, and time to death. Statistical significance was defined as *p* < 0.05. All analyses were performed using Stata v.17.0 (College Station, TX).

## Results

One hundred thirty-five patients were identified on initial screening. After excluding patients without confirmed trauma (*n* = 6) or radiographically confirmed thrombus (*n* = 17), we entered 112 patients into our final analysis. Over the 9-year inclusion period, roughly 10,000 patients (~ 1,100 per year) were admitted to our trauma center with a diagnosis of TBI, leading to an estimated tVST diagnosis rate of 1.12%.

### Demographic and Injury Characteristics

Demographic and injury information is shown in Table 1. Patients were predominantly male (76.8%) and had a mean age of 44 years (min–max: 14–93). Mild (GCS scores 13–15), moderate (GCS scores 9–12), and severe TBI (GCS scores 3–8) was found in 60.7%, 12.5%, and 26.8% of our cohort, respectively. The median admission GCS score was 14 (IQR 8–15).

**Table 1:** Patient characteristics and injury profile

Variable	*N* = 112
Admission characteristics	
Age (years), mean (SD, min–max)	44.4 (19.4, 14–93)
Female sex	26 (23.2%)
Admission GCS, median (IQR)	14 (8–15)
13–15	68 (60.7%)
9–12	14 (12.5%)
3–8	30 (26.8%)
Anticoagulant/antiplatelet use	14 (12.5%)
Leukocytosis (white blood cell count ≥ 12 × 10^9^/L)	47 (42%)
Injury profile	
Trauma type	
Blunt trauma	107 (95.5%)
Penetrating trauma	5 (4.5%)
Skull fracture location	
Frontal bone	19 (17%)
Temporal bone	53 (47.3%)
Parietal bone	62 (55.4%)
Occipital bone	60 (53.6%)
Skull base	37 (33%)
Depressed skull fracture	17 (15.2%)
Dural venous sinus location	
Superior sagittal sinus	36 (32.1%)
Transverse ± sigmoid sinus (lateral sinus)	66 (58.9%)
Sigmoid sinus only	46 (41.1%)
Internal cerebral veins	0 (0%)
Torcula	6 (5.4%)
Multiple sinuses (lateral + superior sagittal)	12 (10.7%)
Degree of venous sinus occlusion	
Complete	23 (20%)
Partial	49 (44%)
External compression	49 (44%)
Initial imaging modality	
CT venography	59 (52.7%)
CT angiography with delayed venous-phase contrast	48 (42.9%)
Follow-up imaging modality	
CT venography	29 (25.9%)
CT angiography with delayed venous-phase contrast	8 (7.1%)
MR venography	15 (13.4%)
TBI-related intracranial hemorrhages	
Epidural hematoma	72 (64.3%)
Acute subdural hematoma	94 (83.9%)
Traumatic subarachnoid hemorrhage	89 (79.5%)
Cerebral contusion	80 (71.4%)
Intraparenchymal hematoma	54 (48.2%)
Intraventricular hemorrhage	13 (11.6%)

Continuous variables are expressed as mean (SD, range) or median (IQR), and categorical variables are expressed as *n* (%)

CT, computed tomography, GCS, Glasgow Coma Scale, IQR, interquartile range, MR, magnetic resonance, TBI, traumatic brain injury

The most frequent mechanisms of injury were ground-level fall (25.9%, *n* = 29), assault (23.2%, *n* = 26), fall from height (18.8%, *n* = 21), pedestrian vs. automobile (15.2%, *n* = 17), bicycle/scooter/skateboard vs. automobile (8.9%, *n* = 10), and motor vehicle collision (0.9%, *n* = 1). Eight patients (7.1%) were found unresponsive with unknown mechanisms of injury.

Traumatic venous sinus thrombosis was diagnosed on vascular imaging within 24 h of hospital arrival in 100 patients (89.3%). Fifty-nine patients (52.7%) underwent CTV, and 48 patients (42.9%) underwent CTA with delayed venous-phase timed contrast. Five patients (4.5%) underwent CT scanning but had incomplete data regarding intravenous contrast timing.

**Fig. 1:**
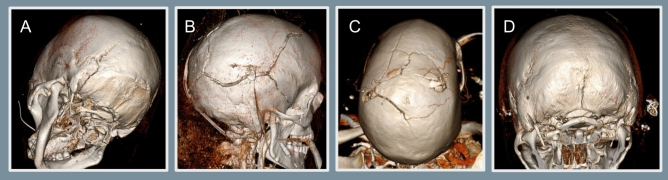
Representative skull fractures. Representative skull fractures from blunt and penetrating head trauma are shown, each fracture associated with venous sinus thrombosis. **a**, A 76-year-old female victim in pedestrian vs. automobile accident with fractures of the left occipital bone and skull base was found to have thrombus in the left transverse sinus. **b**, A 40-year-old man who sustained a gunshot wound to the head suffered skull fractures of the right parietal, occipital, and temporal bones, as well as thrombosis of the right transverse venous sinus. **c**, A 30-year-old man, also a victim of a gunshot wound to the head, was found to have skull fractures of the left and right parietal bones across the vertex, as well as superior sagittal venous sinus thrombosis. **d**, A 23-year-old woman who fell down the stairs and sustained a skull base fracture was found to have thrombus at the confluence of the sinuses (torcula)

One hundred nine patients (97.3%) had skull fractures on initial imaging. Traumatic venous sinus thrombosis was predominantly located near a skull fracture (Fig. 1). Fractures were observed most frequently in the parietal bone (55.4%), occipital bone (53.6%), temporal bone (47.3%), and skull base (33%). Fractures were seen least commonly in the frontal (17.0%) and orbital bones (15.2%). Intracranial hemorrhage was identified in 110 (98.2%) patients. Ninety-four patients (83.9%) had subdural hemorrhage, 89 patients (79.5%) had subarachnoid hemorrhage, and 72 patients (64.3%) had epidural hemorrhage. Cerebral contusion was noted in 80 patients (71.4%).

**Fig. 2:**
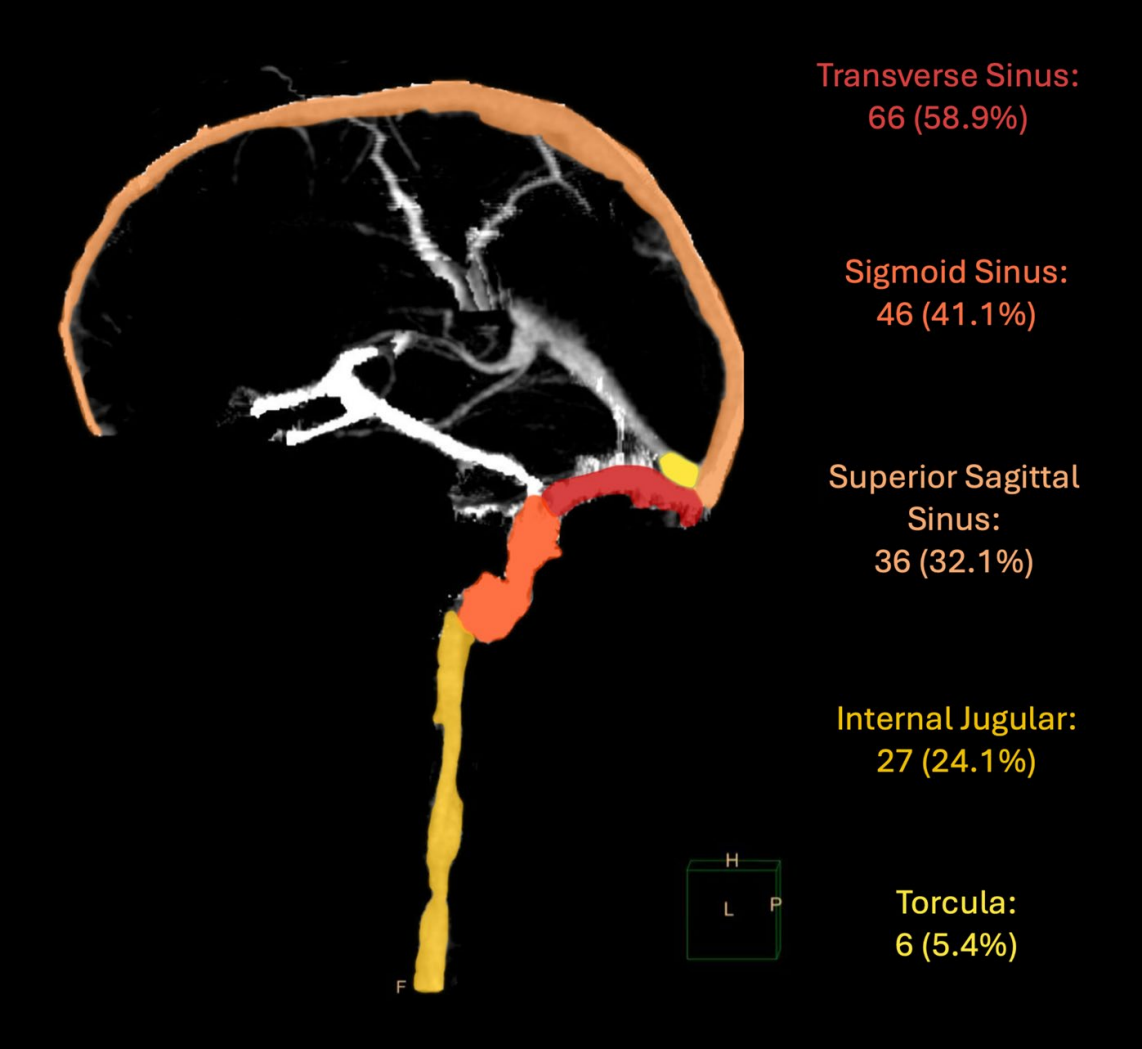
Traumatic venous sinus thrombosis heat maps. Sagittal volume-rendered CT venography projection of heat map based on tVST location frequency. CT computed tomography, tVST traumatic venous sinus thrombosis

The overall distribution of tVST is shown in Fig. 2. Traumatic venous sinus thrombosis was found most frequently in the transverse (58.9%) and sigmoid sinuses (41.1%), with similar frequency between right and left sides. Thirty-six patients (32.1%) developed tVST in the superior sagittal sinus, and six patients developed thrombus in the torcula (5.4%). Four patients with torcula involvement presented with GCS scores ≤ 8. We found no cases of internal cerebral vein thrombosis. Thrombus extended into the internal jugular vein in 27 patients (24.1%). Fifty patients (44.6%) developed tVST in two or more venous sinuses. Twelve patients (10.7%) were found to have thrombus in both superior (superior sagittal sinus) and lateral (transverse or sigmoid) sinuses. These patients presented with a lower median GCS score than the overall cohort (7 vs. 14, IQR 3–8 vs. 8–15).

### Clinical Characteristics and Treatment Differences

Leukocytosis was present on admission in 47 patients (42%). Dehydration was documented in only nine patients (8%). Treatments for VST, ICP elevation, and seizures are shown in Table 2. Nineteen patients experienced seizures, 52.6% of which had focal semiology. Ninety-eight patients (87.5%) received 7 days of seizure prophylaxis per our TBI protocol, and 19 patients (17%) continued anticonvulsant therapy beyond the prophylaxis period for active seizure management. Intracranial pressure monitoring was performed in 58 patients (51.8%) with either an EVD catheter, parenchymal ICP sensor, or both. Seventeen patients required interventions for refractory ICP elevations, of whom 94.1% had multiple tVST. Hyperosmolar therapy was administered to 25.0% of patients. Thirty patients (26.8%) underwent cerebrospinal fluid diversion via EVD, and ten patients (8.9%) required decompressive surgery.

**Table 2:** Interventions and treatments

Indication/intervention	*N* = 112
Venous sinus thrombosis management	
AC	15 (13.4%)
AP therapy	54 (48.2%)
Conservative management (no AC or AP)	58 (51.8%)
ICP management	
Hyperosmolar therapy	28 (25%)
EVD placement	30 (26.8%)
Parenchymal ICP monitor placement	28 (25%)
Surgical decompression	10 (8.9%)
Seizure management	
Prophylaxis (7 days)	98 (87.5%)
Treatment (continued after 7 days)	19 (17%)

Categorical variables are expressed as *n* (%)

AC, anticoagulation, AP, antiplatelet, EVD, extraventricular drain, ICP, intracranial pressure

Fourteen patients were prescribed antithrombotic therapy (AT) prior to admission (12 on aspirin, 1 on rivaroxaban, and 1 on apixaban). Fifty-four patients (48.2%) were initiated on AT during their hospital admissions. Fifteen patients (13.4%) received therapeutic systemic AC, and 39 patients (34.8%) received AP medications. The other 58 patients (51.8%) were managed conservatively with hydration and symptom management. Most of the AP group was prescribed daily low-dose aspirin (81 mg), except for one patient who was prescribed full-dose aspirin (325 mg) for unclear reasons and two patients who were prescribed clopidogrel because of an aspirin allergy. The timing of AT initiation was decided by the neurosurgery attending physician and ranged anywhere from 2 to 14 days post injury. At least four patients were instructed to start taking aspirin 7 days post injury after hospital discharge. Eight patients were prescribed AP as a bridge to AC, and two were switched from AP to AC after tVST propagation was discovered on repeat imaging. Three patients were initiated on AC for reasons other than tVST (two for DVTs, one for pulmonary embolism). The median GCS scores were 10 (IQR 7–14) in the AC group, 14 (10.5–15) in the AP group, and 14 (7.2–14) in the conservative management group. Supportive therapy with intravenous fluid was documented in only 32 patients (28.6%), but this may be an underestimate given the higher number of patients in the conservative management group.

### Clinical and Radiographic Outcomes

Mortality occurred in 23 patients (20.7%), 16 of whom (69.6%) arrived with GCS scores ≤ 8. Withdrawal of life-sustaining therapies was noted for 16 (69.6%) deceased patients. Cause of death was missing for three patients. Patients who survived to hospital discharge were younger, more often male, and had a higher GCS score on arrival. In a Cox proportional hazards model, AT treatment was associated with a higher chance of survival to hospital discharge (hazard ratio [HR] 0.29, 95% confidence interval [CI] 0.12–0.73). Anticoagulant treatment yielded the strongest, though not statistically significant, association with survival to hospital discharge (hazard ratio [HR] 0.18, 95% confidence interval [CI] 0.02–1.32). Antiplatelet treatment was also associated with a higher likelihood of survival to hospital discharge (hazard ratio [HR] 0.337, 95% confidence interval [CI] 0.13–0.90). Hazard ratios for additional covariates were as follows: age, hazard ratio (HR) 1.04 (95% confidence interval [CI] 1.02–1.06); female sex, hazard ratio (HR) 1.12 (95% confidence interval [CI] 0.45–2.80); and emergency department (ED) GCS, hazard ratio (HR) 0.78 (95% confidence interval [CI] 0.71–0.85). Because of the small number of events spread across three treatment groups, we did not examine multivariable models. A comparison of outcomes by treatment group is shown in Table 3.

**Table 3:** Patient characteristics and outcomes by treatment strategy

Variable	AC (*n* = 15)	AP (*n* = 39)	Conservative (*n* = 58)
Age (years), mean (SD)	43.2 (19.1)	42.3 (18.2)	46.1 (20.4)
Female sex	5 (33.3%)	9 (23.1%)	12 (20.7%)
GCS on arrival, median (IQR)	10 (7–14)	14 (10.5–15)	14 (7.25–14)
≤ 8	7 (46.7%)	7 (17.9%)	16 (27.6%)
9–12	1 (6.7%)	6 (15.4%)	7 (12.1%)
13–15	7 (46.7%)	26 (66.7%)	35 (60.8%)
VST location			
SSS	4 (26.7%)	10 (25.6%)	22 (37.9%)
Lateral (transverse ± sigmoid) sinus	13 (86.7%)	31 (79.5%)	21 (36.2%)
Both SSS and Lateral sinuses	3 (20%)	3 (7.7%)	6 (10.3%)
Torcula	3 (20%)	1 (2.6%)	2 (3.4%)
Recanalization rates	20%	35.9%	15.5%
Recanalization rates (surviving)	21.4%	38.9%	23.1%
Associated intracranial hemorrhage			
Epidural hematoma	9 (60%)	23 (59%)	40 (69%)
Acute subdural hematoma	11 (73.3%)	35 (89.7%)	48 (82.8%)
Traumatic subarachnoid hemorrhage	11 (73.3%)	32 (82.1%)	46 (79.3%)
Cerebral contusion	9 (60%)	31 (79.5%)	40 (69%)
Intraparenchymal hemorrhage	12 (80%)	13 (33.3%)	29 (50%)
Intraventricular hemorrhage	1 (6.7%)	4 (10.3%)	8 (13.8%)
Surgical ICP management			
EVD placement	9 (60%)	8 (20.5%)	13 (22.4%)
Parenchymal ICP monitor placement	9 (60%)	6 (15.4%)	13 (22.4%)
Surgical decompression	7 (46.7%)	6 (15.4%)	10 (17.2%)
Treatment (AC/AP) initiated for VST	12 (80%)	33 (84.6%)	–
Treatment not for VST (e.g., AC for PE/DVT, AP for arterial dissection)	3 (20%)	6 (15.4%)	–
Bleeding complications	3 (20%)	5 (12.8%)	2 (3.4%)
HR (95% CI)	4.70 (0.78–28.16)	3.08 (0.60–15.87)	
Alive at discharge	14 (93.3%)	36 (92.3%)	39 (67.2%)
HR (95% CI)	0.18 (0.02–1.32)	0.34 (0.13–0.90)	
Withdrawal of life-sustaining treatment, *n* = 16 (% of treatment group)	1 (6.7%)	2 (5.1%)	13 (22.4%)

Continuous variables are expressed as mean (SD) or median (IQR), and categorical variables are expressed as *n* (%). HRs with 95% CIs were calculated using Cox regression models with no ties

AC, anticoagulant, AP, antiplatelet, DVT, deep venous thrombosis, EVD, extraventricular drain, GCS, Glasgow Coma Scale, HR, hazard ratio, ICP, intracranial pressure, IQR, interquartile range, PE, pulmonary emboli, SSS, superior sagittal sinus, VST, venous sinus thrombosis

Hospital LOS ranged from 0 to 174 days, with a median of 6 days (IQR 3–14). We did not observe any association between LOS and survival to hospital discharge (HR 0.98, 95% CI 0.95–1.01). The median LOS was 24 days (IQR 3–43) for AC-treated patients, 10 days (IQR 3–16) for AP-treated patients, and 4 days (IQR 2–9) for conservatively managed patients. Forty-nine patients (43.8%) were discharged home, and 20 patients (17.9%) were discharged to a rehabilitation facility. Seventeen patients (15.2%) were transferred to their in-network health care institutions because of repatriation agreements. Privately insured patients did not return to our system for outpatient care, resulting in very limited follow-up data.

Our study was not powered to determine the statistical significance of bleeding complication rates between tVST management strategies, and a likelihood ratio test of time to bleeding complication across the three treatments was not significant (*p* = 0.18). We observed more bleeding complications in AT-treated patients (HR 3.53, 95% CI 0.75–16.66). However, intrinsic differences between treatment groups made this observation difficult to interpret. Patients who were too severely injured to initiate AT because of ongoing intracranial or systemic bleeding and patients whose families opted for WOLST were more likely to receive conservative management. Bleeding occurred in three patients on AC (21.4%; HR 4.70, 95% CI 0.78–28.16), five patients on AP (12.8%; HR 3.08, 95% CI 0.60–15.87), and two patients (3.4%) receiving conservative management. Female sex was associated with a higher bleeding risk (HR 3.57, 95% CI 1.03–12.39). The most frequent bleeding complication was gastrointestinal bleeding (*n* = 6), followed by new/worsened intracranial hemorrhage (*n* = 3; including one patient who required a decompressive hemicraniectomy as a result). The remaining bleeding events were attributed to other trauma-related injuries.

Follow-up imaging was available at varying time intervals for 52 patients. Imaging was available at 30 days post injury for only 23 patients (AC, 5 patients; AP, 12 patients; no AC/AP, 6 patients). Follow-up imaging consisted of CT or MR imaging with venous-phase timed contrast. Twenty-nine patients (25.9%) underwent CTV, 8 patients (7.1%) underwent CTA with delayed venous-phase contrast, and 15 patients (13.4%) underwent MR venography. Traumatic venous sinus thrombosis recanalization was noted in 15 patients (28.9%) by 30 days post injury and in 26 patients (50%) by 6 months post injury. Among the 15 patients with tVST recanalization by 30 days post injury, 3 were treated with AC, 9 were treated with AP, and 3 were treated with conservative management.

Within the AC group, 14 patients (93.3%) survived to hospital discharge, and 3 patients (20%) had thrombus recanalization at 6 months. Within the AP group, 36 patients (92.3%) survived to hospital discharge, and 14 patients (35.9%) had thrombus recanalization at 6 months. Within the conservative management group, 39 patients (67.2%) survived to hospital discharge, and 9 patients (15.5%) had recanalization by 6 months. After excluding patients who did not survive, we noted recanalization by 6 months in 21.4% of AC-treated patients, 38.9% of AP-treated patients, and 23.1% of conservatively managed patients.

## Discussion

Traumatic venous sinus thrombosis is an important clinical sequela of TBI that relates to all three components of Virchow’s Triad: endothelial injury, hypercoagulability, and venous stasis. Immediately following traumatic injury, endothelial and sympathetic mechanisms activate inflammatory cascades that induce a transient hypercoagulable or hypocoagulable state. This is followed by a more persistent prothrombotic, hypercoagulable state [16, 17]. The hypercoagulable state begins within the first 48 h of trauma and can last for several days before usually resolving by the 2-week mark [17]. Some studies suggest that blunt injuries of moderate severity can cause a prothrombotic state, whereas injuries with hemorrhagic shock can trigger a hypocoagulable state [16]. Trauma induced coagulopathy is dynamic and may vary based on a patient’s unique injuries [16]. Immobility as a result of TBI and/or critical illness is also an independent risk factor for thrombosis. Volume depletion in severe injuries, as well as compression of venous structures by depressed skull fractures, epidural hematomas, or applied cervical collars, can cause venous stasis [4]. In our study, 47% of patients had leukocytosis on hospital arrival, suggesting a state of inflammation and/or dehydration. Though we did not have specific biomarkers available for analysis, the serum levels of inflammatory biomarkers in the presence of tVST may be an interesting topic for future investigation.

The natural history of tVST remains an ongoing area of study because of the relatively low incidence and possible underreporting of tVST [5, 18, 19]. In one systematic review, 4% of patients with TBI who presented to the emergency department were found to have concomitant tVST. This rate increased to 26% when selection was narrowed to patients who had skull fractures that were adjacent to a venous sinus [4]. A separate study showed highest to lowest tVST risk when fractures involved the petrous, temporal, occipital, parietal, and frontal bones [20]. Our findings mirrored this, as most of our patients developed tVST near fractures in the parietal and occipital bones and skull base. Though the incidence of tVST ranges widely (0.3–26.2%) in the literature, our tVST diagnosis rate of 1.12% is similar to estimates from prior single-institution studies [4, 21].

Areas of ongoing study include identifying which patients are at highest risk of developing tVST and understanding how we should treat them. A conservative “watch and wait” approach with intravenous hydration may be reasonable, with escalation to AT if thrombus persists or propagates on serial imaging [22]. A recent retrospective analysis found higher radiographic thrombus recanalization rates and lower mortality rates among patients with tVST who were treated with AC compared to conservative management [12]. The authors found no overall increase in bleeding risk; however, patients with bleeding complications were more likely to have initiated AC earlier. Notably, patients with severe intracranial pathologies were excluded from this study. A different case series demonstrated higher bleeding risk with AC [11].

Traumatic venous sinus thrombosis treatment guidelines do not yet exist. Anticoagulating tVST in patients with TBI can be controversial because of coexisting intracranial hemorrhages and other body trauma. Traumatic venous sinus thrombosis treatment approaches vary widely between hospitals [12, 23]. Decisions regarding tVST surveillance, antithrombotic timing, antithrombotic choice, and treatment duration are often left to provider discretion [10–13]. Interestingly, spontaneous resolution of tVST has been observed in patients who were not initiated on AT [20]. In a recent study by Ma et al., 22% of patients who were not treated with AC or AP therapy showed complete recanalization on follow-up imaging [24]. In our study, 23% of surviving patients who were not treated with AC or AP therapy experienced spontaneous recanalization on follow-up imaging. This was similar to the rate of spontaneous recanalization in another study that examined both anticoagulated and non-anticoagulated patients with tVST [5].

Antiplatelet therapy was the second most common treatment strategy in our study. There is minimal evidence to support AP use in VST. An early tVST study used urokinase and aspirin, but AP therapy for VST fell out of favor after numerous studies demonstrated successful VST recanalization with systemic AC [23]. The American Heart Association/American Stroke Association guidelines recommend 3 to 6 months of therapeutic AC for the treatment of spontaneous, non-traumatic VST [9]. The guidelines do not comment on aspirin and other AP medications. To our knowledge, no studies have directly compared the treatment outcomes of AC vs. AP therapy for VST.

In our study, the absolute number of recanalized patients with tVST was highest in the AP-treated group. However, this group underwent more interval imaging and had fewer patients lost to follow-up. The higher recanalization rates in patients treated with AP therapy was most likely the result of selection bias and lower disease severity. Given the inherent differences between patients in the AC and conservative groups, the similarity in recanalization rates among surviving patients (21.4% and 23.1%) is interesting but should be interpreted with caution. The same can be said for the associations that we observed between AC/AP treatment and survival to hospital discharge. These associations are purely descriptive and do not imply causation. Instead, such associations highlight the presence of confounding by indication. This is most noticeable in the conservative management group, in which patients who were too severely injured (or deceased) did not receive AC or AP therapy.

Here we describe one of the largest cohorts of patients with tVST in the literature. Our study depicted a unique heterogeneity in patient characteristics and management decisions at our institution. However, our study had multiple additional limitations. Patients who were too severely injured to initiate AT because of ongoing intracranial or systemic bleeding and patients whose families opted for WOLST remained in the conservative management (no AC or AP) group. Even though most patients (81.2%) who underwent WOLST were in the conservative management group, the median GCS score of the conservative group was 14 (with a wide IQR of 7.25–14). This suggests a high degree of heterogeneity in this group.

We did not have a standardized institutional protocol to guide timing or modality choice for tVST surveillance imaging. In cases in which VST was assessed first with CTV but later with MR venography, intrinsic differences between imaging modalities may have caused inaccurate assessments of thrombus size or venous sinus recanalization. A portion of our patients underwent CTA with venous-phase timed contrast, which was usually sufficient to evaluate venous sinus pathological findings, but differences in contrast timing between CTA and CTV present a possible limitation to our study.

Another limitation in our study was the incomplete follow-up imaging and data available for our patients, due in part to insurance limitations and transfer agreements with surrounding hospital systems. Many of our patients arrived as trauma activations but transferred back to their home medical institutions once their trauma-related injuries were stabilized. As a result, we may not have fully captured the natural history of tVST within our limited follow-up data. Long-term survival and functional outcome measures, for example, a 6-month Glasgow Outcome Scale, as Ma et al. reported in a recent publication, would have been valuable data to collect [24].

Lastly, our ability to detect and measure associations between treatment strategy and outcome was limited by sample size, the presence of confounders, and a retrospective study design. The bleeding complication rates in our study were difficult to interpret because of wide CIs and inherent differences between treatment groups. Both AC and AP groups had high rates of survival to hospital discharge (93.3% and 92.3%, respectively). Did this truly reflect a prolonged benefit with these treatment strategies? These numbers may suggest a selection bias toward administering AT in patients who have milder intracranial injuries. Our conservatively managed group had lower survival rates, but the majority (81.2%) of patients who underwent WOLST did not receive AC or AP. The conservatively managed group had the most patients with initial GCS scores ≤ 8. We presented data on observed WOLST rates for each tVST treatment strategy, which to our knowledge has not been reported in the tVST patient population. A recent study demonstrated no difference in WOLST rates between TBIs with and without tVST; however, they did not stratify by treatment modality [21]. Although the reasons for WOLST were not captured in our data set, these findings suggest that tVST treatment decisions may be influenced by perceived outcomes based on injury severity. More studies are needed to better understand the optimal management of tVST among heterogeneous groups of patients with TBI.

## Conclusions

Traumatic venous sinus thrombosis is an underrecognized entity that can exacerbate ICP issues and worsen TBI outcomes. In this retrospective descriptive study, we highlighted significant heterogeneity in tVST monitoring and treatment practices at our level one trauma center. We noted similar tVST recanalization rates among treatment modalities and more bleeding complications in patients treated with AT. However, our findings most importantly showcased inherent differences between tVST treatment groups, presence of selection bias, and clear confounding by indication. Initial disease severity and providers’ predictions of clinical outcomes likely influenced whether patients with tVST at our institution received AP, anticoagulant, or conservative therapy. Our study highlights the need for more investigation. Management of tVST remains an area of ongoing research, with additional studies needed to adequately power future analyses.
